# Impact of the COVID-19 Pandemic on Patient Flow in the Nuclear Medicine Department of a COVID-19 Reference Hospital in Greece

**DOI:** 10.7759/cureus.93492

**Published:** 2025-09-29

**Authors:** Georgia Vazoura, Georgios Giakoumettis, Alexandra Kriari, Evanthia Giannoula, Paraskevi Exadaktylou, Ioannis Iakovou, Emmanouil Papanastasiou

**Affiliations:** 1 Laboratory of Computing, Medical Informatics, and Biomedical-Imaging Technologies, School of Medicine, Aristotle University of Thessaloniki, Thessaloniki, GRC; 2 Medical Physics and Digital Innovation Laboratory, American Hellenic Educational Progressive Association (AHEPA) University Hospital, School of Medicine, Aristotle University of Thessaloniki, Thessaloniki, GRC; 3 Second Academic Nuclear Medicine Department, American Hellenic Educational Progressive Association (AHEPA) University Hospital, School of Medicine, Aristotle University of Thessaloniki, Thessaloniki, GRC

**Keywords:** collective dose, covid-19, diagnostic imaging, nuclear medicine department, patient flow

## Abstract

Background: The COVID-19 pandemic has influenced medical practices globally, including nuclear medicine (NM). This study aimed to assess the impact of the pandemic on patient flow in the Nuclear Medicine Department of the American Hellenic Educational Progressive Association (AHEPA) University Hospital, a COVID-19 reference center in Greece, by comparing the yearly number of examinations and collective doses (CDs) from 2019 to 2023.

Materials and methods: Our Department keeps detailed records of all activities administered for diagnostic and therapeutic procedures. Administered activities for pediatric patients are calculated using the European Association of Nuclear Medicine (EANM) pediatric dosage card. According to the International Atomic Energy Agency (IAEA) recommendations, administered activities for adult patients are calculated based on the patient’s weight. This practice results in similar effective doses for all patients. CDs can thus be estimated by multiplying the typical effective dose per examination by the corresponding number of patients. CDs for the pre-pandemic year (2019), the intra-pandemic period (2020-2021), and the post-pandemic years (2022-2023) were estimated.

Results: A total of 8945 exams were performed during this period (2019: 2486; 2020: 1649; 2021: 1098; 2022: 1484; 2023: 2228). About 6417 (71.7%) were myocardial perfusion (MP) scans, followed by 627 (7%) bone scans. Yearly CDs (in man-sieverts (manSv)) were: 2019: 8.62 manSv; 2020: 6.11 manSv; 2021: 4.93 manSv; 2022: 5.46 manSv; 2023: 8.08 manSv. MP scans were responsible for 64.5% of the CDs, followed by diagnostic iodine-131 (I-131) scans (8.2%), bone scans (7.2%), and iodine-123 (I-123) ioflupane brain single-photon emission computed tomography (SPECT) scans (6.5%). Exams mostly affected by the pandemic (comparing years 2021 and 2019) included dynamic renograms (-88.9%), technetium (Tc)-99m hexamethylpropyleneamine oxime (HMPAO) brain scans (-83.6%), and Tc-99m thyroid scans (-77.5%). On the other hand, the number of Tc-99m macroaggregated albumin (MAA) lung perfusion scans increased by 70.6%. Most exams only partially recovered in the last post-pandemic year.

Conclusion: The COVID-19 pandemic significantly impacted the number of diagnostic exams in our Department. The most significant decline occurred in 2021, with a 55.8% reduction in the number of exams and a 42.9% decrease in CD compared to pre-pandemic levels. Two years later, the patient flow has not yet fully recovered to the pre-COVID-19 levels.

## Introduction

The COVID-19 pandemic has significantly affected European healthcare systems, disrupting routine medical practices and straining resources [1]. Hospitals, clinics, and other healthcare facilities rapidly adapted to the challenges brought about by the virus, leading many departments to experience significant reductions in patient flow, with nuclear medicine (NM) departments being no exception [2,3]. The pandemic affected nearly every area of medicine, with resources being redirected towards treating COVID-19 patients, resulting in delays and cancellations of routine medical procedures [4]. Diagnostic NM, which relies on examinations such as myocardial perfusion (MP) scans, bone scans, brain scans, and dynamic renograms, faced unique challenges during the pandemic [5,6]. The reduced availability of healthcare services, fear of infection, and the overwhelming focus on COVID-19 patients collectively contributed to a sharp decline in routine diagnostic procedures [5].

NM plays a critical role in diagnosing and managing a wide range of conditions, including cardiovascular diseases, malignancies, and neurological disorders [7]. It involves the use of radioactive tracers to monitor the function of organs and tissues, offering valuable insights into disease progression and treatment efficacy. The routine examinations in NM departments, such as myocardial perfusion imaging (MPI), bone scans, and thyroid scans, rely heavily on steady patient inflow to maintain clinical operations and ensure timely diagnosis [6].

However, the onset of the pandemic brought numerous challenges, requiring hospitals to adapt in order to continue providing essential services quickly [8]. The need for strict infection-control measures, the redeployment of medical staff and facilities for COVID-19 care, and patients' hesitancy to visit hospitals due to fear of contracting the virus all contributed to a significant variation in patient flow. Non-urgent diagnostic procedures were often postponed [9]. This reduction in NM activity was further worsened by travel restrictions, which limited patients' access to specialized diagnostic centers [10].

In the context of NM, the collective dose (CD) is one of the key metrics affected by the pandemic. A variation in the number of diagnostic procedures inevitably leads to a variation in CDs. By analyzing CDs, it is possible to assess how the reduction in patient flow affected the overall functioning of NM departments [11].

This study aimed to assess the impact of the COVID-19 pandemic on patient flow and CDs in the Nuclear Medicine Department of the American Hellenic Educational Progressive Association (AHEPA) University Hospital, a COVID-19 reference center in Greece. By collecting and analyzing examination data from 2019 to 2023, the extent to which the pandemic affected the number of diagnostic and therapeutic procedures conducted can be determined. Furthermore, due to the protocols for calculating administered activities used in the Department, which result in approximately weight-independent effective doses for each type of examination, an assessment of the CDs delivered during these years due to NM diagnostic scans can be performed.

## Materials and methods

This study was conducted at AHEPA University Hospital, a major healthcare facility in Thessaloniki, Greece, designated as a COVID-19 reference hospital during the pandemic. As a reference center, the AHEPA Hospital played a crucial role in managing COVID-19 patients while continuing to provide essential medical services, including diagnostic imaging through its Nuclear Medicine Department. During the study period, the Nuclear Medicine Department of AHEPA Hospital was equipped with one dual-head single-photon emission computed tomography (SPECT) gamma camera (Discovery NM630, General Electric Healthcare, Chicago, IL).

Since 2000, the Department has been using in-house developed software to assist medical physicists and NM technologists in the preparation of technetium (Tc)-99m radiopharmaceuticals. The software performs all the necessary calculations concerning molybdenum (Mo)-99/Tc-99m generator elutions, labeling of radiopharmaceuticals, radioactivity concentrations, and individual administrations, taking into account radioactive decay. It also helps to calculate administration activities according to patient weight and keeps detailed records of all administered activities for diagnostic examinations. The same software is also used to record all the other radionuclides that are used in the "hot lab," to calculate the necessary volumes to be withdrawn for each administration, to record all the patients administered activities, and finally to calculate the necessary storage time of the radioactive waste prior to safe disposal.

These detailed records were used to collect data for all the diagnostic examinations performed at the Nuclear Medicine Department from 2019 to 2023. This five-year period was divided into the pre-pandemic year (2019), the intra-pandemic years (2020-2021), and the post-pandemic years (2022-2023) in order to assess the impact of the pandemic on NM practices at AHEPA Hospital. Data collected included examination type and administered activity for each patient. No patient identification data were collected. For each year, the total number of examinations conducted was calculated for each examination type.

The Department follows a strict protocol for calculating the radiopharmaceutical activities administered for each examination type for pediatric and adult patients. The 2016 version of the European Association of Nuclear Medicine (EANM) dosage card is used to ensure appropriate activity levels for pediatric patients [12,13]. For adult overweight patients, the International Atomic Energy Agency (IAEA) guidelines for activity escalation were applied [13]. This consistent approach ensured that effective doses for each examination type remained comparable across patients with different body weights, allowing for a reasonably accurate estimation of CDs [14].

The CDs (in man-sieverts (manSv)) for each diagnostic examination type for each year between 2019 and 2023 were calculated by multiplying the effective dose per examination type by the corresponding number of patients undergoing each examination each year [11]. Changes in CDs over time were analyzed to assess the impact of the COVID-19 pandemic. Therapeutic administrations were not considered.

Statistical analysis was performed to compare the number of diagnostic exams and CDs across the pre-pandemic, intra-pandemic, and post-pandemic years. Descriptive statistics were used to summarize the data, including the total number of exams performed each year and the corresponding CDs. Percent changes in patient flow and CDs were calculated to quantify the variation during the pandemic years and the extent of recovery in the post-pandemic period. Additionally, the variation in specific types of diagnostic exams was analyzed to determine which procedures were most affected by the pandemic. No statistical hypothesis testing was performed. The analysis was limited to descriptive statistics, with results expressed as counts (N) and percentage changes between the compared years.

## Results

During the five years from 2019 to 2023, the Nuclear Medicine Department of AHEPA University Hospital performed 8945 diagnostic and 298 therapeutic procedures. These included Tc-99m sestamibi MP scans, Tc-99m methylene diphosphonate (MDP) bone scans, Tc-99m thyroid scans, Tc-99m hexamethylpropyleneamine oxime (HMPAO) brain perfusion scans, Tc-99m macroaggregated albumin (MAA) lung perfusion scans, Tc-99m diethylenetriamine pentaacetic acid (DTPA) dynamic renograms, Tc-99m sestamibi parathyroid scans, I-123 ioflupane scans, and whole body iodine-131 (I-131) scans after therapeutic administrations for treatment of differentiated thyroid cancer (DTC) and hyperthyroidism. The total number of diagnostic and therapeutic procedures performed each year is presented in Table 1.

**Table 1:** Total number of diagnostic and therapeutic procedures from 2019 to 2023, presented as counts (N) and percentages (%).

Year	Diagnostic procedures (N)	Diagnostic procedures (%)	Therapeutic procedures (N)	Therapeutic procedures (%)
2019	2486	98.11	48	1.89
2020	1649	96.89	53	3.11
2021	1098	94.57	63	5.43
2022	1484	95.74	66	4.26
2023	2228	97.04	68	2.96

**Figure 1:**
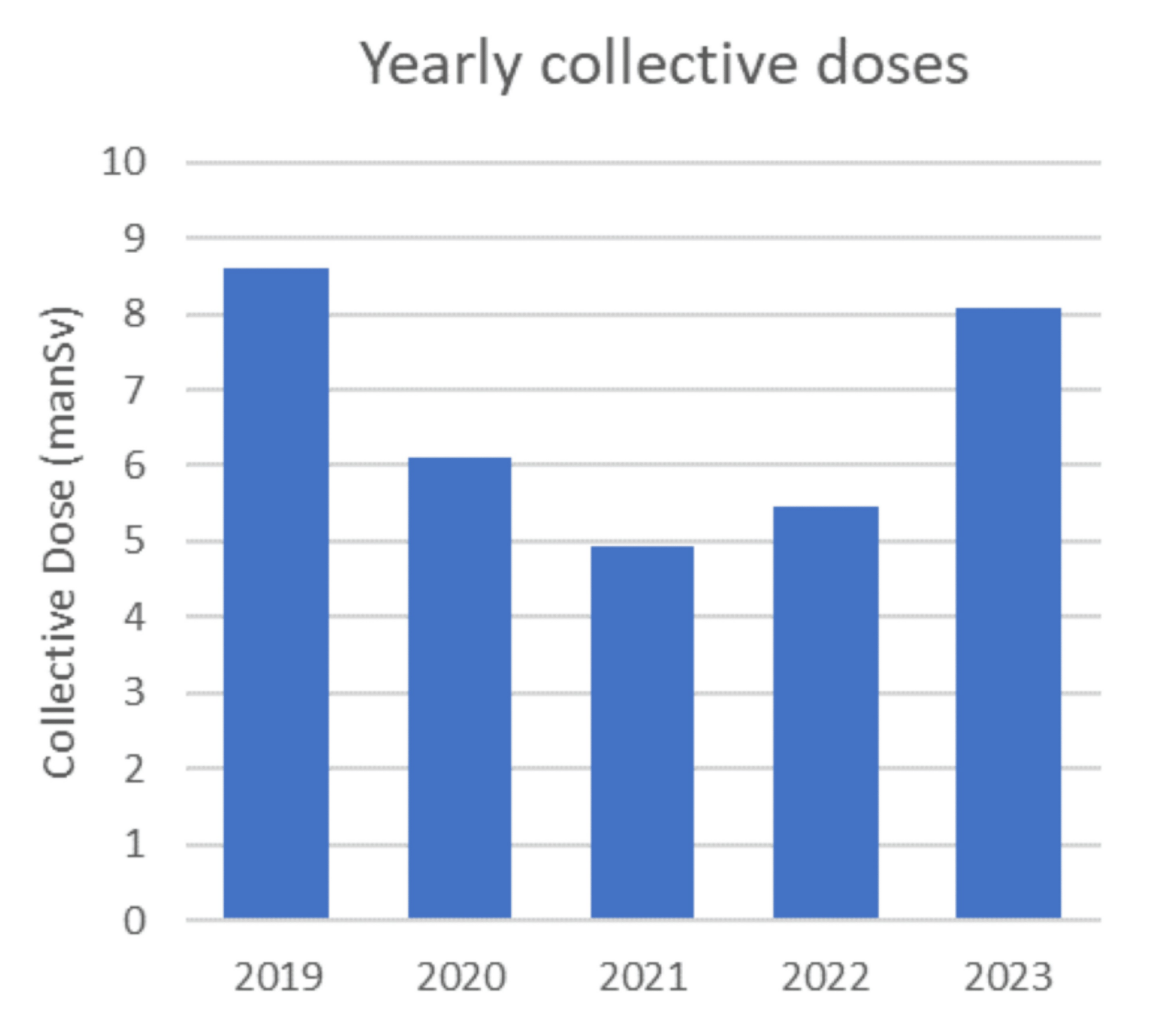
Yearly collective doses from 2019 to 2023, presented as counts (N). manSV: man-sieverts

The total CDs, which were 8.62 manSv in 2019, decreased significantly during the pandemic years and reached 4.93 manSv in 2021 (-42.9%). CD recovery initiated in 2022 (5.46 manSv) and continued during 2023 (8.08 manSv) (Figure 1); nevertheless, it remained slightly below the pre-pandemic levels (-6.3%).

**Figure 2:**
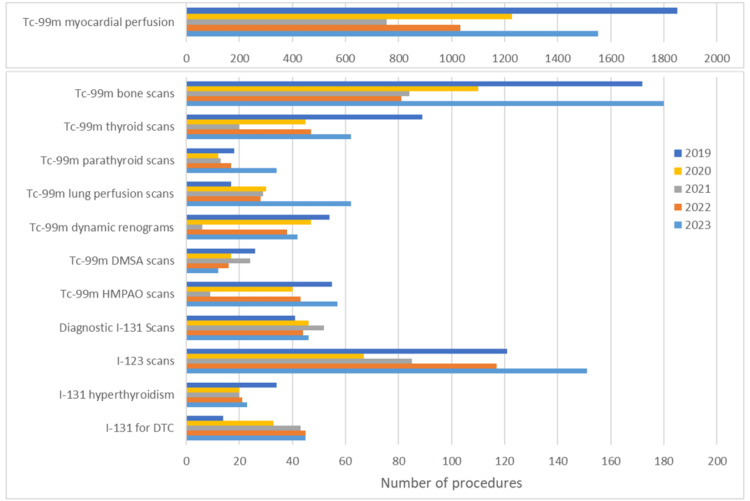
Number of nuclear medicine procedures from 2019 to 2023, presented as counts (N). Tc: technetium; I-131: iodine-131; I-123: iodine-123; HMPAO: hexamethylpropyleneamine oxime; DMSA: dimercaptosuccinic acid; DTC: differentiated thyroid cancer

Figure 2 presents a grouped bar chart illustrating the annual number of diagnostic and therapeutic procedures performed in the Nuclear Medicine Department between 2019 and 2023. MP scans showed a marked decline during the pandemic years, reaching their lowest point in 2021. Although a recovery was observed in 2022 and 2023, the number of procedures remained below pre-pandemic levels. A similar trend was observed for bone scans, thyroid scans, HMPAO brain scans, and dynamic renograms, which experienced substantial reductions in 2020 and 2021, followed by a progressive increase in the subsequent years. Notably, HMPAO brain scans and dynamic renograms demonstrated a sharp rebound in 2022, recovering from the significant drop observed in 2021. Lung perfusion scans deviated from this pattern, with a sharp increase in 2020. This was followed by relatively stable values and a second increase in 2023.

Regarding therapeutic procedures, I-131 administrations for DTC treatment increased during 2020 and 2021 and remained stable through 2023. In contrast, I-131 hyperthyroidism treatments declined in 2020 but showed a gradual recovery over the following years.

The annual CDs per procedure from 2019 to 2023, along with the corresponding percent annual variations, are presented in Table 2. The highest decrease in CDs between 2020 and 2019 was observed for Tc-99m thyroid scans (-49.4%), followed by I-123 scans (-44.6%). The only procedures that showed an increase in CDs between 2020 and 2019 were Tc-99m lung perfusion scans (76.5%) and diagnostic I-131 scans (8.2%). Tc-99m dynamic renograms were the procedure with the highest annual decrease in CD during 2021 (-85.3%), followed by Tc-99m HMPAO scans (-77.5%) and Tc-99m thyroid scans (-55.6%). During 2022, most procedures had started to recover, with Tc-99m dynamic renograms and Tc-99m HMPAO scans showing the highest increase in CDs (487.7% and 377.8%, respectively). The general recovery continued in 2023, with Tc-99m bone scans and Tc-99m lung perfusion scans showing the highest increase in CDs (122.2% and 121.4%, respectively). Tc-99m dimercaptosuccinic acid (DMSA) renal scans and diagnostic I-131 scans were the only procedures that showed an annual decrease in CD during 2023 (-25.0% and -11.2%, respectively).

**Table 2:** Annual collective doses (manSv) per procedure (N) and corresponding percent annual change (%) relative to the previous year (shown in parentheses). Tc: technetium; MP: myocardial perfusion; SPECT: single-photon emission computed tomography; I-131: iodine-131; I-123: iodine-123; HMPAO: hexamethylpropyleneamine oxime; DMSA: dimercaptosuccinic acid; MIBI: sestamibi; manSV: man-sieverts

Procedure	2019	2020	2021	2022	2023
Tc-99m MP	5918.0	4222.5 (-28.6%)	2833.0 (-32.9%)	3337.5 (17.8%)	5113.5 (53.2%)
Tc-99m MP (stress)	2720.0	1668.0 (-38.7%)	922.0 (-44.7%)	1498.0 (62.5%)	2208.0 (47.4%)
Tc-99m MP (rest)	3198.0	2554.5 (-20.1%)	1911.0 (-25.2%)	1839.5 (-3.7%)	2905.5 (58.0%)
Tc-99m bone scan	653.6	418.0 (-36%)	319.2 (-23.6%)	307.8 (-3.6%)	684.0 (122.2%)
Tc-99m thyroid scan	284.8	144.0 (-49.4%)	64.0 (-55.6%)	150.4 (135%)	198.4 (31.9%)
Tc-99m HMPAO scan	357.5	260.0 (-27.3%)	58.5 (-77.5%)	279.5 (377.8%)	370.5 (32.6%)
Tc-99m dynamic renogram	62.7	49.6 (-20.9%)	7.3 (-85.3%)	42.9 (487.7%)	50.6 (17.9%)
Tc-99m DMSA renal scan	41.6	27.2 (-34.6%)	38.4 (41.2%)	25.6 (-33.3%)	19.2 (-25.0%)
Tc-99m lung perfusion	27.2	48.0 (76.5%)	46.4 (-3.3%)	44.8 (-3.5%)	99.2 (121.4%)
Tc-99m parathyroid MIBI scan	104.4	69.6 (-33.3%)	75.4 (8.3%)	98.6 (30.8%)	197.2 (100%)
I-123 SPECT	532.4	294.8 (-44.6%)	374.0 (26.9%)	514.8 (37.6%)	664.4 (29.1%)
Diagnostic I-131 scan	487.0	527.0 (8.2%)	643.9 (22.2%)	569.3 (-11.6%)	505.8 (-11.2%)

Therapeutic I-131 administrations for DTC treatment increased by 135.7% in 2020 and by 30.3% in 2021, followed by minor changes in 2022 (4.6%) and 2023 (0%). I-131 administrations for hyperthyroidism treatment decreased by 41.2% in 2020, then saw a slight increase of 5% in 2022, followed by a further increase of 9.5% in 2023.

All Tc-99m procedures decreased by 33.1% in 2020 and 39.4% in 2021, followed by increases of 40.4% and 54.6% in 2022 and 2023. I-123 and I-131 procedures decreased by 21% in 2020 and increased by 20.5%, 13.5%, and 16.7% in the subsequent years.

The diagnostic examinations most significantly affected by the pandemic (as compared to 2019) included Tc-99m DTPA dynamic renograms (-88.9%), Tc-99m HMPAO brain perfusion scans (-83.6%), and Tc-99m thyroid scans (-77.5%). On the contrary, the number of Tc-99m MAA lung perfusion scans increased by 70.6% from 2019 to 2021. Tc-99m sestamibi MP scans constituted the majority of exams performed during this period, accounting for 71.7% of the total exams. Although the number of MP scans declined during the pandemic years, they remained the predominant procedure performed. Bone scans represented 7% of the total exams, and they followed a similar trend, with a significant decrease during the pandemic (Table 3).

**Table 3:** Variation in the number of procedures between 2021 and 2019, presented as percentages (%) and counts (N). Tc: technetium; SPECT: single-photon emission computed tomography; I-131: iodine-131; I-123: iodine-123; HMPAO: hexamethylpropyleneamine oxime; MDP: methylene diphosphonate; MAA: macroaggregated albumin; DTPA: diethylenetriamine pentaacetic acid; DTC: differentiated thyroid cancer

Nuclear medicine procedure	2021 vs 2019 (%)	Number of procedures in 2021 (N)	Number of procedures in 2019 (N)
Tc-99m sestamibi myocardial perfusion SPECT imaging	-59.2%	755	1852
Tc-99m MDP bone scans	-51.2%	84	172
Tc-99m thyroid scans	-77.5%	20	89
Tc-99m HMPAO brain perfusion SPECT imaging	-83.6%	9	55
Tc-99m MAA lung perfusion scans	+70.6%	29	17
Tc-99m DTPA dynamic renograms	-88.9%	5	39
Tc-99m sestamibi parathyroid scans	-27.8%	13	18
I-123 SPECT imaging	-29.8%	85	121
I-131 whole body scans	+26.8%	52	41
I-131 administrations for DTC	+207.1%	43	14
I-131 administrations for hyperthyroidism	+41.2%	20	34

## Discussion

A study by Kirienko et al. (2022) highlighted the extensive impact of the COVID-19 pandemic on healthcare systems, particularly with severely reduced routine medical procedures. Healthcare resources were redirected to COVID-19 patient care, which caused delays in non-urgent medical interventions, including diagnostic imaging [2].

Vaz et al. (2023) pointed out that hospitals were overwhelmed by COVID-19 patients, which resulted in critical shortages of medical supplies, hospital beds, and staff. This crisis underscored the essential need for effective pandemic response strategies [15].

According to a study by Paez et al. (2022), the pandemic underscored the importance of NM and other diagnostic services in managing not only COVID-19-related respiratory complications but also the backlog of different conditions. However, many diagnostic services were postponed, leading to delayed diagnoses for conditions like cardiovascular diseases. This delay has potential long-term effects on patient outcomes, making it essential for healthcare systems to prioritize recovery efforts, enhance diagnostic capacities, and implement strategies to address postponed or missed examinations [16].

The COVID-19 pandemic significantly impacted the operations of the Nuclear Medicine Department at AHEPA University Hospital, particularly in terms of patient flow and CDs administered. The results of this study show a significant decline in both the number of diagnostic exams and the corresponding CDs during the pandemic's peak, with the most significant reductions occurring in 2021. During this year, patient flow dropped by 55.8% and CDs decreased by 42.9% compared to pre-pandemic levels in 2019.

While there has been a partial recovery in 2022 and 2023, patient flow and CDs have not fully returned to the levels observed before the pandemic. Specific examinations, such as dynamic renograms and brain scans, experienced dramatic reductions. In contrast, lung perfusion scans increased, reflecting the increased need for lung-related diagnostics due to the respiratory effects of COVID-19. MPI is an essential non-invasive cardiac imaging test crucial in diagnosing cardiovascular diseases [17]. The significant decrease in these scans during the pandemic, especially in 2021, likely reflects the postponement of non-urgent cardiovascular procedures. This could have impacted patients with undiagnosed or untreated coronary artery disease, potentially leading to delays in essential care. The partial recovery observed in 2022 and 2023 highlights the gradual return of routine cardiac diagnostics as pandemic pressures diminished. Similarly, bone scans, primarily used for detecting fractures or malignant diseases [18], also experienced a decline during the pandemic. This reduction may be attributed to the delayed diagnosis of bone diseases. The significant recovery in 2023, with a 122.2% increase compared to 2021, indicates that a delay in diagnostic imaging may have been resolved as healthcare systems began to stabilize.

In the case of Tc-99m thyroid scintigraphy, the pandemic's effects were more noticeable. These scans are a diagnostic technique that provides a complete view of the entire thyroid gland in a single image [19]. They experienced one of the most significant reductions during the pandemic. This reduction may reflect the prioritization of urgent care over routine thyroid monitoring. The sharp recovery in thyroid scans in 2022 suggests that, as the pandemic eased, there was an increased demand for thyroid diagnostics, likely driven by postponed evaluations.

Likewise, Tc-99m HMPAO brain scans, which assess cerebral perfusion and can aid in diagnosing neurological conditions like stroke or dementia [20], saw a steep decline in 2021. This decline in 2021 might reflect the postponement of routine neuroimaging, while the dramatic recovery (+377.8%) in 2022 suggests a resurgence in neurological diagnostics, possibly addressing delayed assessments from earlier in the pandemic. In a similar pattern, dynamic renograms, used for evaluating renal function [21], drastically decreased during the pandemic. This drop could be attributed to the reprioritization of non-urgent renal evaluations. The significant increase in 2022 (+487.7%) indicates a return to normal levels of renal diagnostics, reflecting the resumption of postponed evaluations.

Furthermore, Tc-99m lung perfusion is a nuclear imaging test that utilizes a perfusion scan to map blood flow distribution within the lungs [22]. The increase in Tc-99m lung perfusion scans during the pandemic reflects the heightened demand for respiratory diagnostics in response to COVID-19-related complications. Specifically, there was a 76.47% increase from 2019 to 2020 and a 70.6% rise in 2021 compared to pre-pandemic levels, highlighting how the Nuclear Medicine Department adapted to evolving clinical needs. This underscores the important role of nuclear imaging in the assessment and management of pulmonary conditions during the pandemic.

Similarly, parathyroid MIBI (sestamibi) scans, often used for diagnosing hyperparathyroidism [23], experienced moderate fluctuations during the pandemic. The slight reduction in 2021 might indicate a deprioritisation of endocrine diagnostics, but the recovery by 2023 suggests that routine endocrinological care gradually resumed as healthcare systems adapted.

An important operational observation concerns the source of radiopharmaceuticals. While iodine-123 (I-123) and I-131 were ordered externally for specific patients and remained relatively stable, Tc-99m tracers were produced in-house and exhibited notable fluctuations. These variations likely reflect pandemic-related constraints on local production capacity. The greater consistency in I-123 and I-131 use may be attributed to structured ordering processes and prioritization for specific clinical needs, which made them less susceptible to supply chain disruptions.

I-123 iodobenzamide (IBZM) scans are used for the differential diagnosis between Parkinson's disease and other neurodegenerative diseases [24], while I-123 DaTscan is a technique for Parkinsonian syndrome [25]. The fluctuations in the number of I-123 scans during the pandemic reflect the shifting priorities in diagnostic imaging, with routine thyroid and neuroendocrine assessments likely being delayed and addressed as pandemic pressures subsided.

Alexander and Larsen (2002) demonstrated that high-dose I-131 therapy is a safe, effective, and streamlined approach for the treatment of hyperthyroidism, particularly in patients with Graves’ disease [26]. Despite such evidence supporting the efficacy of this method, the use of I-131 for the treatment of hyperthyroidism was negatively affected by the COVID-19 pandemic, with a noticeable decline during the years 2020-2021. Although there has been a gradual recovery, the 2023 data indicate that activity has not yet fully returned to pre-pandemic levels. This likely reflects delays in diagnosis or changes in therapeutic practice, even though this radioiodine therapy remains an established and efficient treatment option.

Zidan et al. (2004) demonstrated that I-131 ablation therapy for DTC, when tailored to postoperative radioiodine uptake, can achieve high ablation success rates even with reduced doses, supporting a more individualized and efficient treatment strategy. This finding underscores the clinical importance of maintaining access to radioiodine therapy [27]. The use of I-131 therapy for DTC remained relatively stable during the initial years of the COVID-19 pandemic, with only mild fluctuations observed between 2019 and 2021. This suggests that, despite the widespread strain on healthcare services, radioiodine treatment for thyroid cancer was prioritized to a certain extent. However, a slight decline in 2022 followed by only a modest increase in 2023 may reflect lingering challenges in oncology service delivery or delays in surgical referrals and postoperative care. These trends highlight the need to ensure continuity of care for cancer patients during future disruptions, particularly when effective adjuvant therapies like I-131 are available.

The diagnostic I-131 scans after the total thyroidectomy include pre-treatment and post-treatment radioiodine imaging. Pre-treatment scans are used to determine the extent of postoperative thyroid remnants. Post-treatment scans are performed in the patients after radioiodine therapy [28]. The relatively stable pattern in the number of diagnostic I-131 scans throughout the pandemic, with slight fluctuations, suggests that these exams remained a priority despite the broader disruptions in healthcare services. The 12.2% and 13% increases in 2019-2020 and 2020-2021, respectively, indicate that diagnostic assessments continued, perhaps reflecting the critical need for accurate diagnoses in thyroid conditions, where early detection and monitoring are essential. The mild decrease in 2021-2022 suggests a temporary reduction, but the recovery in subsequent years underscores the importance of resuming these diagnostic services as pandemic pressures ease.

Limitations

Limitations must be considered for this type of study. This study was conducted in a single COVID-19 reference hospital. The findings may be more generalizable if data from additional COVID-19 reference hospitals were included. The analysis was based on departmental dose records rather than detailed patient data. Further investigation of detailed patient data could provide the direct impact on patient health outcomes. Finally, factors such as changes in referral patterns, staffing shortages, and interruptions in the radiopharmaceutical supply chain during the pandemic may have influenced the results but were not fully taken into account.

## Conclusions

The findings of this study highlight the persistent effects of the COVID-19 pandemic on the Nuclear Medicine Department and underscore the challenges healthcare providers faced in maintaining diagnostic services during the pandemic. The continued recovery of patient flow and CDs in the post-pandemic period indicates a return to routine activities. Still, it also emphasizes the need for strategies to ensure adaptability in healthcare services during future pandemics or similar crises.
